# Altered Light Conditions Contribute to Abnormalities in Emotion and Cognition Through HINT1 Dysfunction in C57BL/6 Mice

**DOI:** 10.3389/fnbeh.2018.00110

**Published:** 2018-06-08

**Authors:** Yuan Zhou, Hao-kang Zhang, Fei Liu, Gang Lei, Peng Liu, Tong Jiao, Yong-hui Dang

**Affiliations:** ^1^College of Medicine & Forensics, Xi’an Jiaotong University Health Science Center, Xi’an, China; ^2^College of Public Health, Xi’an Jiaotong University Health Science Center, Xi’an, China; ^3^Clinical Research Center of Shaanxi Province for Dental and Maxillofacial Diseases, College of Stomatology, Xi’an Jiaotong University, Xi’an, China; ^4^Department of Psychiatry, First Affiliated Hospital of Xi’an Jiaotong University Health Science Center, Xi’an, China

**Keywords:** circadian rhythms, photoperiods, depression, anxiety, cognition, HINT1, apoptosis

## Abstract

In recent years, the environmental impact of artificial light at night has been a rapidly growing global problem, affecting 99% of the population in the US and Europe, and 62% of the world population. The present study utilized a mouse model exposed to long-term artificial light and light deprivation to explore the impact of these conditions on emotion and cognition. Based on the potential links between histidine triad nucleotide binding protein 1 (HINT1) and mood disorders, we also examined the expression of HINT1 and related apoptosis factors in the suprachiasmatic nucleus (SCN), prefrontal cortex (PFC), nucleus accumbens (NAc) and hippocampus (Hip). Mice exposed to constant light (CL) exhibited depressive- and anxiety-like behaviors, as well as impaired spatial memory, as demonstrated by an increased immobility time in the tail suspension and forced swimming tests, less entries and time spent in the open arms of elevated plus-maze, and less platform site crossings and time spent in the target quadrant in the Morris water maze (MWM). The effects of constant darkness (CD) partially coincided with long-term illumination, except that mice in the CD group failed to show anxiety-like behaviors. Furthermore, HINT1 was upregulated in four encephalic regions, indicating that HINT1 may be involved in mood disorders and cognitive impairments due to altered light exposure. The apoptosis-related proteins, BAX and BCL-2, showed the opposite expression pattern, reflecting an activated apoptotic pathway. These findings suggest that exposure to CL and/or darkness can induce significant changes in affective and cognitive responses, possibly through HINT1-induced activation of apoptotic pathways.

## Introduction

The light/dark cycle provides periodically alternating illumination conditions for life on earth, which helps entrain biological rhythms for individuals (Bedrosian and Nelson, 2013). Nevertheless, with the advent of electrical lighting at the beginning of the 20th century, humans and other species have had to align behavioral and physiological processes to artificial light cycles instead of the natural solar cycle, ultimately leading to disrupted circadian rhythms (Fonken et al., 2009). Urban development has further exacerbated the range of “light pollution,” negatively affecting 99% of the population in the US and Europe, and 62% of the world population (Navara and Nelson, 2007). Previous studies have demonstrated that aberrant light conditions increase susceptibility to heart disease (Ha and Park, 2005), cancer (Schernhammer et al., 2001; Davis and Mirick, 2006), sleep disturbances (Deboer et al., 2007; Kohyama, 2009), as well as major depressive disorder and mood disorders (Dumont and Beaulieu, 2007).

To evaluate the effects of altered illumination on emotional and cognitive behavior, animal models with disrupted circadian rhythms have been established. These models involve constant illumination (Deprés-Brummer et al., 1995; Ikeda et al., 2000), light deprivation (Gonzalez and Aston-Jones, 2008), and altered light periods (de la Iglesia et al., 2004; Einat et al., 2006). Nevertheless, reports on the interaction of long-term illumination with depressive- and anxiety-like responses and cognition have been inconsistent. Several studies using constant light (CL) exposure to disrupt circadian rhythms have demonstrated that depressive-like behavior was present across various species (Fonken et al., 2009; Bedrosian and Nelson, 2013; Tapia-Osorio et al., 2013). In contrast to these findings, Flaisher-Grinberg et al. (2011) demonstrated that altered photoperiods had no harmful effects on depression phenotypes. The effects of continuous light exposure on anxiety-like responses and cognition have also been inconclusive (Castro et al., 2005; Ma et al., 2007). There is controversy in the literature on whether constant darkness (CD) induces aberrant affective and/or cognitive behaviors. Therefore, we aimed to explore the precise influence of altered illumination on mood and cognition, as well as the mechanisms underlying these effects.

Histidine triad nucleotide binding protein 1 (HINT1), a member of the histidine triad protein superfamily, is widely expressed in multiple tissues including the central nervous system (Lima et al., 1996; Klein et al., 1998). A recent human postmortem analysis suggested that HINT1 may be involved in the pathophysiology of mental disorders, including schizophrenia and bipolar disorder (Vawter et al., 2001, 2002, 2004; Elashoff et al., 2007). HINT1 knockout mice with or without chronic restrained stress exhibited decreased depressive-like behavior and increased anxiety-like behavior (Varadarajulu et al., 2011; Garzón-Niño et al., 2017; Sun et al., 2017). Microarray analysis by Abdel Rassoul et al. (2010) in the primate *Microcebus murinus* and our own research (data not published) confirmed that HINT1 was upregulated in aged and cognition-impaired individuals, pointing to a possible relationship between HINT1 and cognition. HINT1 expression was dysregulated in encephalic regions involved in mood and cognition under pathological conditions (Li et al., 2017). Given that HINT1 is highly correlated with the pathophysiological processes of aberrant mood and cognition-related behaviors, it is important to assess whether HINT1 is implicated in abnormal mood and cognition induced by altered illumination conditions.

HINT1 was originally considered a tumor suppressor (Su et al., 2003), and may inhibit tumor growth by initiating relevant apoptotic pathways (Genovese et al., 2012). There is evidence that altered illumination conditions cause neuronal damage to monoamine systems, including increased apoptosis in several brain regions (Gonzalez and Aston-Jones, 2008). Thus, HINT1 may participate in the regulation of mood and cognition via initiation of apoptotic pathways.

The main aim of this study was to explore whether both CL and/or darkness lead to aberrant emotional behaviors and cognition in mice and to elucidate the underlying association between the HINT1 protein and altered light-related psychiatric symptoms. Consequently, we chose to assess the expression of HINT1 in the prefrontal cortex (PFC), nucleus accumbens (NAc), hippocampus (Hip) and suprachiasmatic nucleus (SCN). We also examined apoptotic-related proteins, BAX and BCL-2, to assess how the dysregulation of HINT1 generates aberrant emotions and cognition induced by altered light conditions.

## Materials and Methods

### Experimental Animals and Grouping

Three-week-old C57BL/6 male mice were purchased from the Animal Center of the College of Medicine, Xi’an Jiaotong University (SCXK, Shan 2012-003). Sixty mice were housed in standard cages (26 cm × 18 cm × 13 cm) under a controlled 12-h/12-h light-dark cycle (lights on at 7:00 A.M.) at room temperature (22 ± 1°C) and 60 ± 5% humidity. Four mice were housed in each cage and had free access to water and food. After 3 weeks of feeding, all the animals were randomly assigned to three groups: (1) Normal (N) mice (*n* = 18) remained in a normal light-dark cycle for 4 weeks; (2) CL mice (*n* = 18) were maintained in animal cabinets with the lights on 200–250 (lx for each cage) for 4 weeks; and (3) CD mice (*n* = 16) were maintained in conditions with lights off (0 lx for each cage) for 4 weeks. Mice in the CD group were maintained in a relatively independent non-transparent cabinet where any light pulses were blocked by a black-out cloth. Experimental manipulations involving mice in the CD group were performed by an experimenter wearing night vision spectacles. Before each test, the animals were placed in the laboratory for 30 min to acclimate to the test environment. Animals were weighed weekly. The experimental protocols were in accordance with the Guide for the Care and Use of Laboratory Animals published by the US National Institutes of Health and approved by the Xi’an Jiaotong University Laboratory Animal Administration Committee. All efforts were made to minimize the number of animals used and their suffering.

### Experimental Design

After 4 weeks in different lighting conditions, the mice underwent a battery of behavioral tests to measure anxiety- and depressive-like behaviors as well as cognition. Behavioral tests were performed for the N mice during their corresponding daytime (9:00–15:00, Eastern Standard Time [EST]) and for CL and CD mice at the same clock time as the LD group (9:00–15:00 EST). The performing time was selected according to the secretion of corticosterone (Park et al., 2013; Bhardwaj et al., 2015). Different illumination treatments were maintained for each group. Ten of 18 mice were subjected to behavioral tests occurring in the following order to minimize the effects of stress in the most sensitive tests: open field test (OFT), elevated-plus maze (EPM), tail suspension test (TST) and forced swim test (FST; Crawley, 2006). All other mice were subjected to the OFT and Morris water maze (MWM). The mice were sacrificed immediately following the FST and MWM. Whole brains were removed rapidly only in the MWM subgroup. The SCN, PFC, NAc and Hip were dissected on an ice-cold plate using the mouse brain atlas as a guide (Franklin and Paxinos, 1997).

### Behavioral Tests

#### Open Field Test

Mice were placed individually into an open field chamber (45 × 45 × 45 cm) for 1 h. The test was performed under three different illumination conditions: 25 lx for the N group, 25 lx for the CL group, and 0 lx for the CD group. These lighting conditions were maintained for the next battery of behavioral experiments. The tracks were recorded by a video tracking system (SMART, Panlab SL, Barcelona, Spain). When monitoring the activity of the CD group, an infrared camera was utilized for recordings and the video tracking system was converted to infrared mode. These conditions were maintained for the following behavioral experiments for the CD group. Total distance and distance per 10 min were calculated as indicators of autonomous activity capacity (Walsh and Cummins, 1976; Liu et al., 2014).

#### Elevated Plus Maze

We performed the EPM test as previously described (Pellow et al., 1985; Liu et al., 2014; Chen et al., 2017). Briefly, the apparatus was composed of two open arms (25 × 5 cm) and two closed arms (25 × 5 × 20 cm). Open and closed arms were cross-shaped and the cross-center was a 5 × 5 cm open platform. The maze was raised 50 cm above the ground. Mice were placed on the central platform facing the open arm. During the 6-min test period, several indicators were measured, including: (1) the number of closed arms entries; (2) the number of open arms entries; (3) the time spent in the open arms; and (4) the time spent in the closed arms. We used the percentage of time spent in the open arms out of the total time spent in the open and closed arms and the percentage of open arm entries out of the total arm entries as measures of anxiety. Indicators were scored manually by a trained experimenter.

#### Tail Suspension Test

The TST was performed as previously reported (Steru et al., 1985). Briefly, mice were suspended with a tape 0.75 cm away from the tip of their tails and elevated 50 cm above the laboratory table. An observer blinded to treatment groups counted the immobility time for 6 min by visual observation.

#### Forced Swimming Test

The FST was based on a previously described protocol (Porsolt et al., 1977; Cryan and Holmes, 2005). Mice were placed individually into a Plexiglas barrel (15.8 cm diameter, 24.5 cm height) filled with water to a depth of 10 cm at a temperature of 22 ± 1°C for 6 min. Subsequently, the mice were dried immediately and returned to their home cages. The immobility time was recorded by a trained observer blinded to treatment groups.

#### Morris Water Maze

The apparatus was composed of a circular pool (100 cm in diameter, 50 cm in height, four quadrants based on four equidistant points on the wall), a platform (34 cm in height, 8 cm in diameter and 2 cm below the surface), and the recording system. The pool water temperature was controlled at 22 ± 1°C. The camera was mounted at approximately 1 m directly above the pool to record the motion trajectory in the N and CL groups. An infrared camera was used to monitor animal activity in the CD group. All the required data could be acquired from the video tracking system. The MWM test was based on a previously described protocol (Vorhees and Williams, 2006) and was divided into three phases: adaptation, spatial learning training, and a probe test. The adaptation phase lasted for 1 day (Day 1), in which the mice were required to freely swim in the pool for 60 s without the platform, and their swimming velocity was recorded. Mice received continuous training four times a day for five consecutive days during the spatial learning training phase (Days 2–6). The platform was fixed during this phase. The time for each mouse to locate the platform was recorded as the escape latency. The average escape latency for the four daily training sessions was analyzed. The mouse was held facing the wall of the pool, placed into the water, and monitored for 60 s. If the mouse failed to locate the platform, it was guided to the platform and retained there for 15 s, and the latency was recorded as 60 s. The shortest time interval between the two training sessions was 10 min. In the probe test (Day 7), the platform was removed, and the swimming route and number of platform site crossings were recorded.

### Analysis of Apoptosis-Related Proteins and HINT1

The expression of BAX, BCL-2 and HINT1 were evaluated with western blot analysis. Isolation of four encephalic regions was performed on ice. Samples from the MWM subgroups were lysed in RIPA lysis buffer (Beyotime, Guangzhou, China), and the protein concentration was determined with a BCA protein assay kit (Pierce, Rockford, IL, USA). The protein extracts (30 μg per lane) were separated by SDS-PAGE and then transferred to polyvinylidene difluoride membranes, which were probed with various primary antibodies. After incubation with the appropriate secondary antibodies for 1 h at room temperature, the membranes were treated with ECL reagents (Bio-Rad, Hercules, CA, USA), and the signals were visualized with an Odyssey Imaging System. The expression levels of specific proteins were normalized to those of β-actin. Image Lab software (Bio-Rad) was used for quantification analysis. The following primary antibodies were used: anti-BAX antibody (AF0120, 1:1000, rabbit monoclonal, Affinity Biosciences, Cincinnati, OH, USA), anti-BCL-2 antibody (AF6319, 1:1000, rabbit monoclonal, Affinity Biosciences), HINT1 (ab124912, 1:1000, rabbit monoclonal, Abcam, Cambridge, MA, USA) and β-actin (C4, 1:5000, mouse monoclonal, Santa Cruz Biotechnology, Santa Cruz, CA, USA).

### Statistical Analysis

All data are expressed as mean ± standard error of mean (SEM), using SPSS 13.0 (SPSS Inc., Chicago, IL, USA) for data processing and analysis. Repeated measures tests followed by Bonferroni *post hoc* tests were used to analyze locomotion every 10 min in the OFT and the latency time in the MWM. One-way analysis of variance (ANOVA) and Dunnett *post hoc* tests were performed to analyze the other indices of the OFT and MWM, as well as the indicators of the EPM, TST, FST, and the results of the western blot analysis. *P* values < 0.05 were considered statistically significant.

## Results

### Effects of Constant Light and Darkness on Spontaneous Activity of C57BL/6 Mice

**Figure 1:**
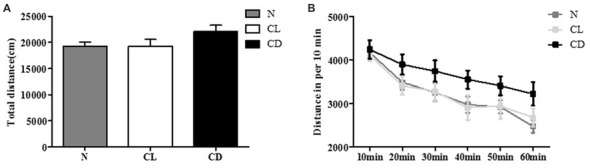
Effects of constant light (CL) and darkness on spontaneous activity of C57BL/6 mice. **(A)** Total distance had no statistical significance among normal (N), CL and constant darkness (CD) group (*P*_A_ = 0.212). **(B)** Distance in per 10 min also had no statistical significance (*P*_B_ = 0.134). *P*_A_ was generated from one-way analysis of variance (ANOVA) test, and *P*_B_ was the value from repeated measurement test of group × phase.

In the OFT, the total distance (cm) and distance per 10 min of C57BL/6 mice receiving different illumination protocols showed no significant difference (*P*_A_ = 0.212, Figure 1A; *P*_B_ = 0.134, Figure 1B). This indicated that the mice had similar spontaneous activity, and provided a movement baseline for the next battery of behavioral experiments (Figure 1).

### The CL and CD Groups Exhibited More Immobility Than Control Mice in TST and FST

**Figure 2:**
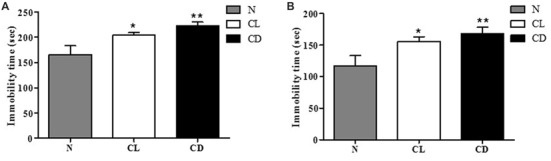
Effect of illumination duration on depressive-like behavior. **(A)** Tail suspension test (TST) manifested that the group of CD and CL had predominantly increasing immobility time compared to the N group (*P*_A_ = 0.006). **(B)** Forced swim test (FST) manifested that the group of CD and CL had predominantly increasing immobility time compared to the N group (*P*_B_ = 0.010). *P < 0.05 represents the statistical significance between N and CL group, **P < 0.01 means significant difference existing between N and CD group.

To examine depression-like behavior in C57BL/6 mice, we conducted two classical behavioral experiments, the TST and FST, choosing total immobility time as an indicator. Mice in the CL and CD groups exhibited predominantly longer immobility time than the mice in the N group (*P*_A_ = 0.006, Figure 2A). This enhancement was more evident in the CD group (*P*_A-CD_ = 0.003) than the CL group (*P*_A-CL_ = 0.049), with an effect size of 0.473 (Cohen’s *d* = 1.074). The FST also produced similar results, demonstrating that immobility time increased in the CL and CD groups (*P*_B_ = 0.010, Figure 2B). Immobility time in the CD group was longer than that in the CL group, with an effect size of 0.230 (Cohen’s *d* = 0.474; *P*_B-CD_ = 0.006; *P*_B-CL_ = 0.038; Figure 2).

### CL Mice Exhibited More Anxiety-Like Behavior Than Controls in EPM

**Figure 3:**
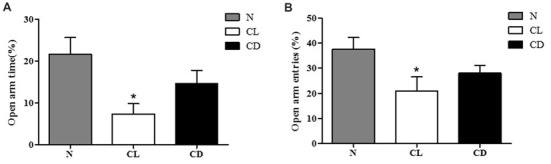
Effect of illumination duration on anxiety-like behavior. **(A)** Times in open arm (%) and **(B)** entries into open arm (%) were significantly declined in CL as compared to N group, while mice in CD group did not apparently exhibited anxiety-like behavior. (*P*_A-CL_ = 0.014; *P*_B-CL_ = 0.237; *P*_A-CD_ = 0.014; *P*_A-CD_ = 0.221). *P < 0.05 represents the statistical significance between N and CL group.

In the EPM, the number of entries and time spent in the open arm (%) were chosen to analyze anxiety-like behavior. Compared to the N group, mice exposed to CL spent significantly less time and had fewer entries into the open arm (*P*_A-CL_ = 0.014, Figure 3A; *P*_B-CL_ = 0.028, Figure 3B). In contrast, there was no significant difference between the N group and CD group in terms of time spent and number of entries into the open arm (*P*_A-CD_ = 0.237; *P*_B-CD_ = 0.221). These results suggest that only CL may induce anxiety-like behavior, while CD has no effect on anxiety-like behavior (Figure 3).

### Exposure to Constant Light or Darkness Impaired Spatial Memory

**Figure 4:**
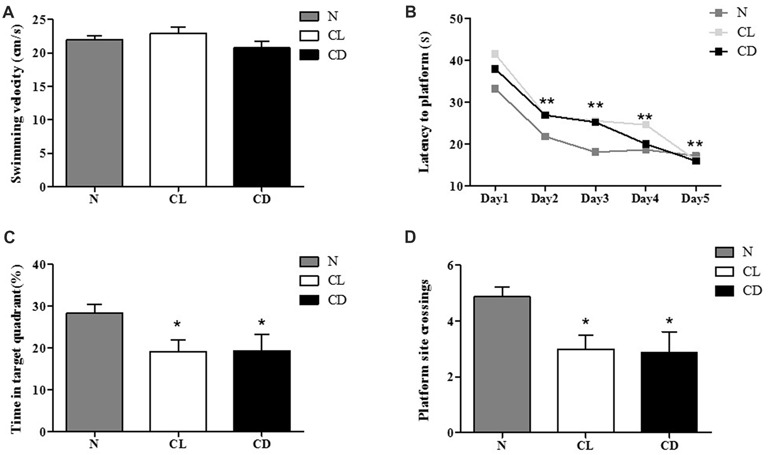
Effect of CL and dark on cognition. **(A)** The mean swimming velocity (cm/s) in the adaptation phase (Day 1) showed no statistical significance (*P*_A_ = 0.216). **(B)** The interaction between time and group presented no significant difference (*P* = 0.635). Escape latency data were averaged across four trials per day for 5 days. Compared to the escape latency in Day 2, this indicator in Day 3–5 significantly decreased (*P* = 0.001), but there was no significant difference among these three groups (*P* = 0.104). **(C,D)** Performance of mice in the probe test (Day 7) appeared that mice exposure to CL and CD had less time in target quadrant and platform site crossings (*P*_C_ = 0.050; *P*_D_ = 0.001). *P < 0.05, **P < 0.01.

For the MWM, there was no significant difference in swimming velocity among all three groups during the adaptation phase without the platform (*P*_A_ = 0.216, Figure 4A). We used these measures as the baseline of locomotor activity for subsequent experiments (Figure 4A). For the spatial learning training, the interaction between time and group showed no significant difference (*P* = 0.635, Figure 4B). The latency time in each group followed a decreasing trend, demonstrating a statistically significant decrease among these time points (*P* = 0.001). However, there was no significant difference among the three groups (*P* = 0.104; Figure 4B). In the probe test, the percentage of time in the target quadrant (%) and the number of platform site crossings in the CL and CD groups was significantly less than that in the control group (*P*_C_ = 0.050, *P*_D_ = 0.032; Figures 4C,D).

### CL and CD Dysregulated the Expression of HINT1 and Apoptosis-Related Proteins in the SCN, PFC, NAc and Hip

**Figure 5:**
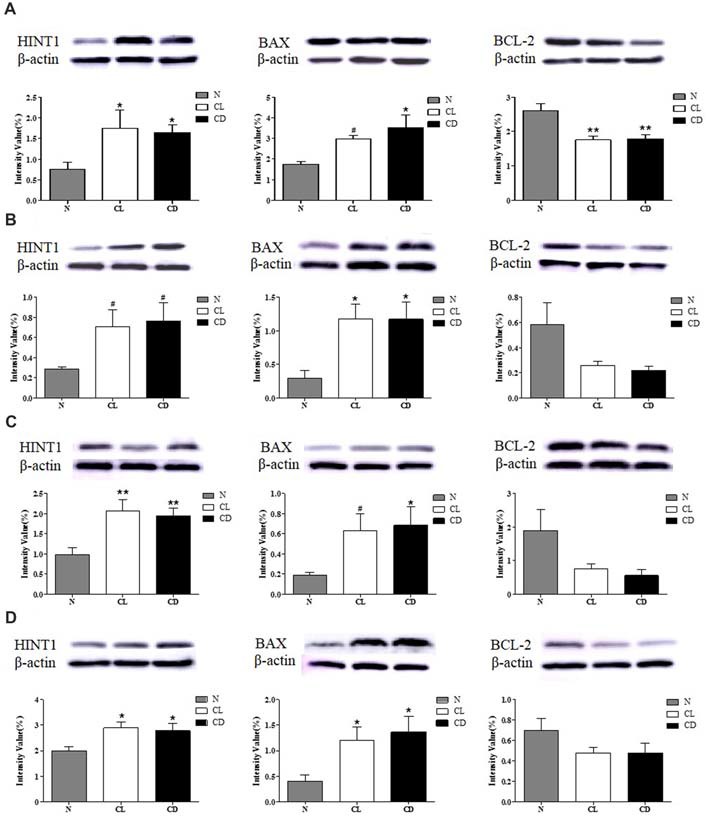
Effect of CL and dark on the expression of histidine triad nucleotide binding protein 1 (HINT1), BAX and BCL-2 in SCN, PFC, NAc and Hip. **(A)** Expression of HINT1, BAX and BCL-2 in SCN (*P*_A1_ = 0.039; *P*_A2_ = 0.028; *P*_A3_ = 0.001); **(B)** Expression of HINT1, BAX and BCL-2 in PFC (*P*_B1_ = 0.110; *P*_B2_ = 0.020; *P*_B3_ = 0.076); **(C)** Expression of HINT1, BAX and BCL-2 in NAc (*P*_C1_ = 0.006; *P*_C2_ = 0.098; *P*_C3_ = 0.082) and **(D)** Expression of HINT1, BAX and BCL-2 in Hip (*P*_D1_ = 0.037; *P*_D2_ = 0.020; *P*_D3_ = 0.189) was determined by Western blotting, where β-actin served as the loading control. The representative images were shown. ^#^*p* < 0.05, *p < 0.05, **p < 0.01.

After the last behavioral test (MWM), the mice were sacrificed and total protein was extracted from the mouse SCN, PFC, NAc and Hip for western blot analysis of HINT1, BAX, and BCL-2 expression. The expression of HINT1 in all four encephalic regions was significantly enhanced after treatment with CL and darkness (*P*_A-HINT1_ = 0.039, Figure 5A; *P*_B-HINT1_ = 0.110, Figure 5B; *P*_C-HINT1_ = 0.006, Figure 5C; *P*_D-HINT1_ = 0.037, Figure 5D), and the change in BAX was consistent with that in HINT1 (*P*_A-BAX_ = 0.028, *P*_B-BAX_ = 0.020, *P*_C-BAX_ = 0.098, *P*_D-BAX_ = 0.020). Conversely, the expression of BCL-2 only showed a decreasing trend in the PFC, NAc, and Hip, but the trend did not reach statistical significance (*P*_B-BCL-2_ = 0.076, *P*_C-BCL-2_ = 0.082, *P*_D-BCL-2_ = 0.189). The amount of BCL-2 protein in the SCN was significantly decreased (*P*_A-BCL-2_ = 0.001; Figure 5).

## Discussion

In the current study, our behavioral experiments showed that chronic illumination results in increased depression- and anxiety-like behaviors and decreased spatial memory. In contrast, CD did not induce anxiety-like behaviors. Moreover, we discovered a significant increase in HINT1 expression and aberrant expression patterns of BAX and BCL-2 in brain regions involved in emotion and cognition, as well as the SCN, which is a key brain region regulating circadian rhythms. Our findings suggest that HINT1 may play a role in aberrant emotion and cognition due to CL and/or darkness by initiating the extrinsic apoptosis pathway.

Our results agree with those of previous studies that have consistently demonstrated an increase in depressive-like responses upon long-term light exposure (Lima et al., 1996; Fonken et al., 2009; Leach et al., 2013; Tapia-Osorio et al., 2013). In addition, exposure to CL was associated with enhanced depression- *and* anxiety-like behaviors, which may partly underlie the co-morbidity of these disorders. Thus, rodent models treated with CL, but not prolonged light, may provide a new perspective to study the induction of depression. Previous research on the association between illumination and anxiety-like behavior has been inconclusive. For example, CL reduced anxiety-like behavior in rats in the EPM (Ma et al., 2007; Fonken et al., 2009), but had no effect on anxiety-related behaviors in a plus-maze discriminative avoidance task (Castro et al., 2005). Nonetheless, our results are consistent with Tapia-Osorio et al. (2013) who reported increased anxiety-like responses after performing CL and CD. A possible reason for these conflicting results is that animals were treated with different light-dark cycles during the experimental adaption phase. Fonken et al. (2009) acclimated mice under a 16:8 light/dark cycle (lights on at 23:00); whereas Tapia-Osorio et al. (2013) used a 12:12 light/dark cycle with lights on at 20:00, and Ma et al. (2007) used a 12:12 light/dark cycle with lights on at 8:00 (Ma et al., 2007; Fonken et al., 2009; Tapia-Osorio et al., 2013). These different protocols may influence the effect of CL and darkness.

The illumination regimens of Fonken et al. (2009) and Tapia-Osorio et al. (2013) are extensively utilized to reverse the natural circadian rhythms of nocturnal animals in order mimic the light/dark cycle of diurnal animals. However, our preliminary experiments (with lights on at 19:00 and off at 7:00) showed that it was inappropriate to reverse the natural circadian rhythms of nocturnal animals, as mice in the control group exhibited anxiety-like behaviors in the OFT (data not published). Although the C57 strain has been used as a background strain for circadian-related targeted mutations (Schubert, 2009; Pezuk et al., 2010), it may introduce confounding factors and limit the discussion of how affective behaviors are influenced by disrupting circadian rhythms in humans. On the other hand, the performing time of behavioral experiment is another factor that may affect behavioral phenotypes induced by different photoperiods. It is generally accepted that all behavioral experiments should be performed during the day (12:12 light/dark cycle), when animals secrete a relatively low level of corticosterone (Lightman et al., 2008). Since the altered photoperiods disrupted the original circadian rhythms of mice in the CL and CD groups, endogenous time markers, such as corticosterone, should be examined to determine when behavioral experiments should be conducted in the CL and CD groups. However, the literature has not yet reached a consensus on the issue of performing time of behavioral experiments. We believe that more standardized and unified protocols should be developed in the future.

The effect of light deprivation on mood remains controversial. We observed apparent depressive-like behaviors in mice undergoing CD, while Tapia-Osorio et al. (2013) only found a trend of depression-like behavior in CD mice, which was of lower intensity than in CL mice and not significantly different from N mice (Tapia-Osorio et al., 2013). Moreover, our findings agree with those of Tapia-Osorio et al. (2013) who showed that CD appeared to have no effect on anxiety-like behavior. CL and light deprivation are two main methods of building animal models with disrupted circadian rhythms (Deprés-Brummer et al., 1995; Ikeda et al., 2000; Gonzalez and Aston-Jones, 2008). However, our results verified that these two different illumination manipulations may induce different behavioral phenotypes, implying that there are different mechanisms involved. There are two main hypotheses for this issue according to the existing literature. One is that an altered photoperiod induces physiological changes directly via disruption of the biological clock function (Ohta et al., 2005). The other is that an altered photoperiod represents a chronic stressor (Ma et al., 2007), which can indirectly affect physiological and behavioral processes (Ling et al., 2009). Indeed, there is evidence that mice exposed to CD maintain rhythmic physiological secretion of melatonin and corticosterone (Tapia-Osorio et al., 2013); therefore, CD may function as a chronic stressor influencing the behavioral phenotype without disputing circadian rhythms.

In terms of cognition, chronic CL and darkness exposure may lead to spatial memory deficits in mice. Our findings are consistent with Ma et al. (2007) who demonstrated that chronic CL induced a slight enhancement of spatial learning ability and negatively impacted spatial memory. In addition, Bedrosian et al. (2013) provided a neural correlate of these behaviors. They demonstrated that hamsters exposed to 4 weeks of CL had reduced dendritic spine density in hippocampal CA1 pyramidal neurons, which is considered to be closely related to learning and memory.

Our data indicate that chronic conditions of CL or CD are also disruptive for HINT1 expression in the SCN, which was significantly enhanced in CL and CD mice. As previously described, disrupted circadian rhythms due to CL and darkness resulted in reduced expression of c-Fos (a marker of neuronal activity) and dysregulated clock genes in the SCN (Salgado-Delgado et al., 2008), implying diminished neuronal activity and altered gene expression profiles in the SCN (Edelstein et al., 2000), suggesting that HINT1 may be implicated. Our unpublished data agree with those of Barbier and Wang (2009) who reported that HINT1 knockout mice demonstrated anti-depressant and anti-anxiolytic behaviors associated with elevated plasma corticosterone levels. Notably, CL resulted in low corticosterone concentration and dysregulation of the hypothalamic-pituitary-adrenal axis (Fonken et al., 2009), which is consistent with altered corticosterone concentration in HINT1 knockout mice. It is likely that corticosterone characterized by rhythmic secretion may be regulated by *HINT1*, similar to the *Per1* gene. Therefore, we speculate that *HINT1* may function as a general clock gene and thereby affect emotional and cognitive disorders induced by disrupted circadian rhythms. However, this study cannot directly verify this speculation, and thus we look forward to further studies that will examine the role of HINT1 in regulating circadian rhythms and will assess the emotional and cognitive correlates of HINT1 dysregulation.

HINT1 was upregulated in the PFC, NAc and Hip, which agrees with our previous research (Li et al., 2017). Li et al. (2017) reported that HINT1 was downregulated in the PFC and NAc but was upregulated in the Hip in socially isolated mice that manifested anxiety- and depression-like behaviors as well as impaired cognition. Although altered light periods and social isolation are different, they can both be considered stressors with different underlying molecular mechanisms. Moreover, thalamic relays of the SCN project to regions directly involved in regulating mood and cognition, such as the PFC, Hip and amygdala, suggesting that altered illumination conditions may negatively influence mood and cognition (Bedrosian and Nelson, 2013). We examined the expression of BAX and BCL-2 expression in four encephalic regions. BAX and BCL-2 belong to apoptotic-promoting and apoptotic-inhibiting classes, respectively (Hanus et al., 2015). HINT1, as a haploinsufficient tumor suppressor, could trigger the extrinsic apoptosis pathway to inhibit tumoral growth (Genovese et al., 2012). For example, in response to the knockdown of the *Hint1* gene by short hairpin RNA, downregulation of p53 and BAX expression was also observed (Yu and Zhang, 2005; Weiske and Huber, 2006). These findings agree with our results, as we observed that BAX was upregulated in parallel with HINT1 in CL and CD groups, in contrast to BCL-2. Based on these findings, we speculate that CL and darkness promote HINT1 expression in specific encephalic regions and may trigger apoptotic pathways, ultimately causing affective disorders and cognitive impairment.

There are some limitations of this study that need to be acknowledged. It is generally accepted that the standard duration of the EPM is 5 min. However, based on the previous results by Chen et al. (2017) and our group, we eventually decided to perform the EPM for 6 min, which should be validated in the future (Xing et al., 2013; Chen et al., 2017). Moreover, although we selected the performing time of the behavioral experiments based on published articles and our preliminary experiment, this decision may have rendered the behavioral results of the CD group incomparable with those of other similar studies. Besides, the lack of an endogenous time marker in this experiment may lead to false positive results in behavioral tests. This study is also limited in explaining whether the changes in behavior and protein expression are due to constant illumination patterns or whether they are a consequence of altered circadian rhythmicity. However, altered photoperiods and disrupted circadian rhythms are not mutually exclusive; conversely, altered photoperiods are the common method used to construct animal models with disrupted circadian rhythms (Tapia-Osorio et al., 2013). Only mice in the MWM subgroup were used to examine HINT1, BAX and BCL-2 expression. We believe that the behavioral experiments may have had a slight influence on the biochemical data but that it would not have covered the effect of treatments (Barbier and Wang, 2009; Li et al., 2017; Sun et al., 2017). However, further biochemical studies should be conducted on mice only receiving CL or darkness exposure, but not behavioral experiments, on the premise of complying with ethics.

In conclusion, this study suggests that altered photoperiods induce depression- and anxiety-like behaviors and impair spatial memory in C57BL/6 mice. Increased HINT1 expression in the SCN after continuous light and dark implies that HINT1 may be associated with circadian rhythms. This is the first attempt to evaluate the underlying relationship between HINT1 and circadian rhythms and provide a basis for future experiments. In encephalic regions closely associated with mood disorders, namely the PFC, NAc and Hip, altered duration of lighting led to upregulated HINT1, accompanied by increased BAX and decreased BCL-2. These molecular correlates point to a mechanism underlying abnormalities in emotional and cognitive behaviors due to disrupted circadian rhythms. We believe that HINT1 may be a potential drug target for emotion-related disorders and a possible circadian rhythm-related gene, but the pathophysiological mechanisms should be further explored.

## Author Contributions

YZ and Y-hD conceived and designed the experiments. YZ, H-kZ, FL and GL performed the experiments. TJ, PL and YZ analyzed the data. YZ and Y-hD wrote the manuscript.

## Conflict of Interest Statement

The authors declare that the research was conducted in the absence of any commercial or financial relationships that could be construed as a potential conflict of interest.

## Abbreviations

ANOVA: analysis of variance
CD: constant darkness
CL: constant light
EPM: elevated-plus maze
FST: forced swim test
HINT1: histidine triad nucleotide binding protein 1
Hip: hippocampus
MWM: Morris water maze
N: normal
NAc: nucleus accumbens
OFT: open field test
PFC: prefrontal cortex
SEM: standard error of the mean
SCN: suprachiasmatic nucleus
TST: tail suspension test.
